# Upper extremity motor outcomes during paired vagus nerve stimulation in chronic ischemic stroke: a retrospective single-health-system cohort study

**DOI:** 10.3389/fneur.2026.1872498

**Published:** 2026-09-30

**Authors:** Ronald Benitez, Michelle Darling, Paige Zaorski, Quinn Galligan, Jack Maurer, Nicole McNamara, Shannon J. Doherty

**Affiliations:** 1 Atlantic Brain and Spine, Morristown, NJ, United States; 2 Atlantic Health, New Providence, NJ, United States; 3 Atlantic Health, Newton, NJ, United States; 4 Atlantic Health, Cedar Knolls, NJ, United States; 5 Mobia Medical, Inc., Austin, TX, United States

**Keywords:** activities of daily living, functional recovery, rehabilitation, stroke, treatment outcome, upper extremity, vagus nerve stimulation, stroke rehabilitation

## Abstract

**Background:**

Persistent upper extremity (UE) impairment after stroke is a major cause of long-term disability, and treatment options in the chronic phase are limited. In randomized trials, vagus nerve stimulation paired with task-specific rehabilitation (Paired VNS) produced greater improvement in UE function and activity than the same rehabilitation with sham stimulation, leading to FDA approval in 2021.

**Methods:**

We conducted a retrospective, observational cohort study of adults with chronic stroke and persistent UE deficits who underwent Vivistim implantation and participated in a Paired VNS program delivered across 5 outpatient clinics within our health system. The primary outcome was change in Fugl-Meyer Assessment–Upper Extremity (FMA-UE) from baseline (Paired VNS therapy initiation) to end of the intended Paired VNS therapy protocol (18 sessions). A ≥6-point improvement defined a clinically meaningful response. Adverse events were a secondary safety outcome, ascertained from routine clinical documentation and not systematically collected beyond early postoperative follow-up.

**Results:**

32 patients underwent device implantation; 28 (88%) completed the intended protocol within the health system and had paired FMA-UE assessments available for the primary analysis. Ages ranged from 34 to 81 years (mean 62.1) and the median time since stroke was 3.0 years (IQR 1.7–4.2). FMA-UE scores improved for all 28 local completers. The mean increase of 9.43 points was statistically significant (95% CI 7.20–11.66; *p* < 0.001), the median increase was 8.0 points (IQR 5.0–13.25) and 20 patients (71.4%; 95% CI 52.9–84.7%) achieved a clinically meaningful response. Chart-derived clinical notes provided illustrative context regarding individual changes in daily activities that accompanied UE motor improvement in selected cases. Among the 32 implanted patients, one postoperative hematoma resolved without intervention and one device was explanted after 410 days due to chest tingling.

**Conclusions:**

In this retrospective single-health-system cohort, Paired VNS was associated with significant improvement in FMA-UE scores among 28 local completers. The findings are directionally consistent with prior trial evidence.

## 1 Introduction

Despite advances in acute stroke management and rehabilitation, stroke remains the third-leading cause of death and long-term disability combined worldwide (1). Many people experience persistent motor deficits into the chronic phase (2), with upper extremity (UE) impairments among the most prevalent and disabling sequelae (3, 4). Historically, substantial recovery was thought to occur only in the first weeks to months after stroke, with minimal improvement expected thereafter (5–7). Consequently, therapeutic options beyond the subacute phase remain limited, and persistent impairments continue to limit independence in activities and participation (8).

Recent developments in neuromodulation have shifted this paradigm. Implantable vagus nerve stimulation (VNS), when paired with task-specific rehabilitation (Paired VNS), has demonstrated meaningful and sustained improvements in UE function years after a stroke (9–13). VNS promotes neuromodulatory signaling associated with neuronal plasticity, which is thought to facilitate the reorganization of motor networks when temporally paired with task-based motor training (14).

A pivotal triple-blinded, randomized, controlled trial (VNS-REHAB), conducted in the United States and the United Kingdom, evaluated the safety and efficacy of Paired VNS (Vivistim^®^ System, Mobia Medical, Inc., Austin, TX; see Figure 1) in patients with UE impairment after ischemic stroke. Participants engaged in 18 sessions of outpatient rehabilitation paired with either active or sham stimulation over six weeks.

**Figure 1:**
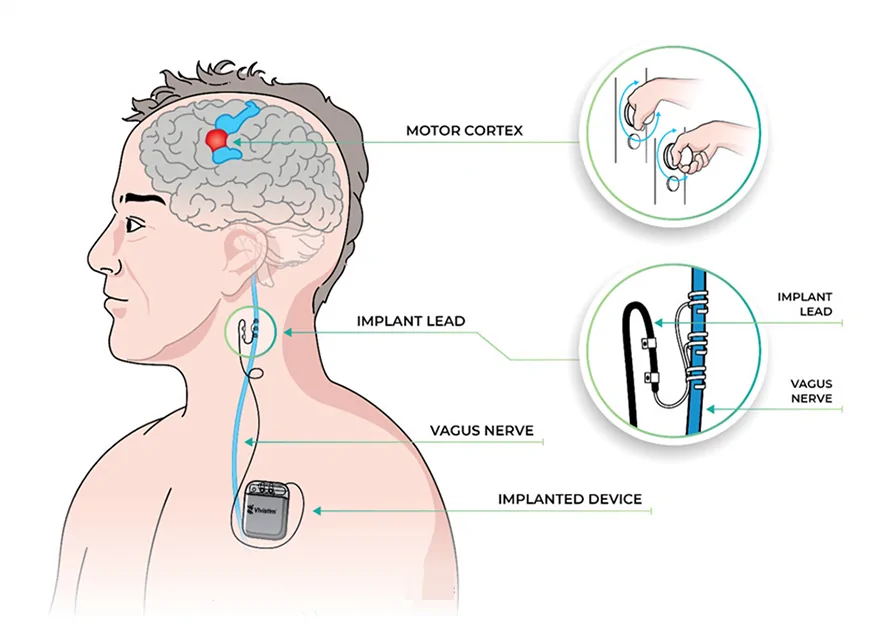
Paired vagus nerve stimulation system. Schematic representation of the implanted Vivistim paired vagus nerve stimulation (Paired VNS) system, including the pulse generator and cervical vagus nerve cuff lead. Image provided by Mobia Medical, Inc. and reproduced with permission. © 2026 Mobia Medical, Inc.

The VNS-REHAB trial demonstrated that Paired VNS resulted in significantly greater and clinically meaningful improvements in UE function and activity compared to sham stimulation, leading to FDA approval in 2021 (9). Long-term follow-up has shown durable treatment effects, with participants maintaining clinically and statistically significant improvements across multiple functional domains and quality of life measures for several years (9, 12, 13, 15).

The objective of this study was to report UE motor outcomes from a cohort of stroke survivors treated with Paired VNS therapy within our health system and to provide selected chart-derived clinical notes as illustrative context.

## 2 Methods

### 2.1 Study design and setting

This was a retrospective, observational cohort study conducted in adults with chronic, post-stroke UE motor impairment who underwent Vivistim^®^ implantation at Overlook Medical Center followed by Paired VNS therapy across five outpatient rehabilitation clinics within the Atlantic Health System.

### 2.2 Participants

Patients were identified via physician referral across stroke neurology, physiatry, and neurosurgery, as well as through affiliated outpatient therapy sites and self-referral. Candidates for Paired VNS therapy were adults with chronic ischemic stroke who had persistent UE motor deficits, were good surgical candidates, and could participate in high-intensity outpatient rehabilitation. Additional program-specific considerations included psychosocial factors that may influence therapy adherence such as motivation and caregiver support.

Clinical eligibility was not determined by strict replication of trial criteria. Within our system, chronicity was considered to be at least 6 months post-stroke, with no predetermined upper limit on time since stroke. Similarly, our program did not apply prespecified FMA-UE cutoffs. FMA-UE scores were considered alongside clinical examination, functional limitations, individualized rehabilitation goals, and the patient's capacity to participate in intensive therapy. Scores of approximately 15–55, together with some active movement of the wrist and at least two digits, were generally considered consistent with potential candidacy, though final eligibility was determined on an individual basis. Accordingly, the observed baseline FMA-UE and time since stroke ranges reflect the program's broader clinical assessment rather than adherence to clinical trial criteria.

Because this study analyzed a retrospective cohort of patients who were referred, screened, and underwent implantation through existing clinical pathways, prospective screening and recruitment data were not systematically captured and were unavailable for reporting in this manuscript.

The primary analysis population comprised implanted patients with both baseline (Paired VNS therapy initiation) and end-of-protocol FMA-UE assessments. Patients who had not completed the therapy protocol by the data cutoff or completed therapy outside the system were excluded from the primary analysis. Adverse events noted during routine postoperative care (1–2 weeks) are reported for all implanted patients. No systematic long-term adverse event monitoring was performed, though any events captured incidentally during unrelated data extraction are reported here.

### 2.3 Exposure

Patients were implanted with the Vivistim^®^ Paired VNS™ System under general anesthesia in an outpatient surgical setting. Implantation followed a standard VNS procedure which has been previously described (16). All device implantations were performed by a single operator with extensive experience in VNS surgery.

Following medical clearance, patients began a structured therapy protocol of Paired VNS combined with task-specific UE rehabilitation. Paired VNS therapy was available at five outpatient clinics within the health system, and patients could attend the location most convenient to them. Each site had occupational therapists (OTs) previously trained to deliver VNS paired with high-intensity, task-specific UE rehabilitation. No study-specific training was provided.

The intended protocol consisted of 18 sessions of 90 min scheduled over approximately six weeks, with flexibility to reschedule due to illness or unforeseen circumstances. During in-clinic sessions, stimulation was delivered by an OT via a wireless remote, synchronized with targeted active movements during UE activities (e.g., reaching for, or grasping utensils). Tasks were individualized to each patient's functional ability and personal goals. Typical stimulation parameters include an output current of 0.8 mA delivered over 0.5 s, a pulse width of 100 μs, and a stimulation frequency of 30 Hz, as established in previous studies (9). Individual patient-level stimulation settings were not retrospectively evaluated in this study. Patients were also instructed to self-activate their devices outside of the clinic to initiate cyclical VNS while engaging in their structured home exercise program and activities of daily living (12). Self-activated VNS sessions are typically 30 min, performed up to 8 times a day, with a daily maximum of 4 h (17). Data regarding home self-activation, therapy dose, and fidelity metrics were not evaluated as part of this study.

### 2.4 Data collection

Retrospective data collection was conducted via patient chart and clinical note review. September 25, 2025, served as the data cutoff for this analysis. Two research associates extracted the data, which were verified by the principal investigator for accuracy and completeness. Therapy notes were reviewed and summarized by three OTs.

### 2.5 Outcomes

The primary outcome was the change in FMA-UE score from baseline (Paired VNS therapy initiation) to the end-of-protocol. End-of-protocol FMA-UE assessments were conducted at the 18th session, approximately 6 weeks after therapy initiation. One patient completed the final session and assessment after approximately 7 weeks as therapy was temporarily interrupted by illness. No prespecified assessment window was applied as treatment and assessments occurred as part of routine clinical care. The FMA-UE assessments were administered by OTs with prior clinical training and experience in motor assessment, without any additional study-specific training. OT assessors also delivered the intervention and documented clinical therapy notes as a part of routine clinical care. As a result, assessments were unblinded and data regarding inter-rater reliability are unavailable. Adverse events were recorded as secondary safety outcomes. Adverse-event determination was based on routine clinical documentation and was not systematic beyond early postoperative follow-up. Three patients continued Paired VNS beyond the intended protocol period and had additional FMA-UE assessments at 5 months, 14 months, and 22 months, respectively. Their longer-term outcomes are not included in the primary analysis but are reported descriptively as exploratory observational findings.

### 2.6 Analysis

Descriptive statistics were used to summarize patient characteristics and adverse events. Continuous variables were reported as means with standard deviations (SD) and medians with interquartile ranges (IQR), as appropriate; categorical variables were summarized with counts and percentages. Baseline and end-of-protocol FMA-UE scores were compared using a paired-samples *t*-test. The mean within-participant change and corresponding 95% confidence interval (CI) were calculated using the *t*-distribution and the standard error of the mean difference. Statistical significance was defined as a two-sided *p* value < 0.05. The normality of within-participant difference scores was assessed using the Shapiro-Wilk test. Because the difference scores showed modest positive skewness, a percentile bootstrap 95% CI for the mean difference (10,000 resamples with replacement; random seed 12,345) was calculated as a sensitivity analysis.

An improvement of ≥6 points on the FMA-UE was used to define a clinically meaningful response, consistent with the responder convention used in the pivotal VNS-REHAB trial (9). This threshold falls within previously reported anchor-based minimum clinically important difference (MCID) estimates of 4.25–7.25 points for different facets of UE movement and function in chronic stroke (18). The responder proportion was reported with a 95% CI using the Wilson score method. Cohen's *d*_*z*_ was calculated as a standardized descriptive measure of within-participant change. Baseline FMA-UE scores were additionally categorized using a four-level impairment severity classification derived by cluster analysis in chronic stroke applying cut-points for the 33-item assessment including reflex items (19). The four impairment levels applied by FMA-UE range were: severe (0–15), severe-moderate (16–34), moderate-mild (35–53), and mild (54–66).

Analyses were conducted using available complete cases, and missing data were not imputed. Descriptive statistics and the paired-samples *t*-test were performed using IBM SPSS Statistics, version 30. The percentile bootstrap CI and the Wilson score CI for the responder proportion were computed in Python 3.11.3 using NumPy and SciPy.

### 2.7 Ethics statement

This retrospective review of existing clinical records was undertaken to evaluate the outcomes of an established clinical program. The activity was submitted through Atlantic Health System's Research Self Certification process on November 9, 2025, and acknowledged on March 3, 2026. Based on the information submitted and the investigator's determination that the activity did not meet the regulatory definition of human subjects research, IRB review and approval were not required. The acknowledgment does not constitute an IRB review, approval, or formal determination by the IRB. Because the activity was not considered human subjects research, the federal regulations governing IRB oversight and informed consent did not apply to the project as described. Accordingly, separate informed consent for research participation was not required. All patients had previously provided informed consent for device implantation and rehabilitation as part of routine clinical care.

All data were de-identified before analysis by assigning unique study IDs, removing direct identifiers, and storing the linkage file on an encrypted, access-restricted server. Additional individual patient-specific consent was gathered for handwriting samples.

### 2.8 Reporting guideline

This study is reported in accordance with the Strengthening the Reporting of Observational Studies in Epidemiology (STROBE) guidelines. A completed STROBE checklist is provided as Supplementary material (20).

## 3 Results

### 3.1 Participants

The device was implanted in 32 patients between February 23, 2023, and August 8, 2025. Three patients completed the Paired VNS protocol at outside institutions after unrelated medical issues delayed rehabilitation (seizures related to changes in epilepsy medication, an arm injury sustained in a fall, and diplopia that impaired driving ability). Two of these individuals resided out of state and transitioned to clinics closer to home once medically stable; the patient with transportation limitations transitioned to home-based Paired VNS delivered by an independent mobile therapist. Outcome data were unavailable for all three because follow-up occurred outside the initial health system. One additional patient remained mid-protocol at the time of analysis and did not yet have an end-of-protocol FMA-UE assessment for comparison. FMA-UE outcomes were therefore analyzed for 28 local protocol completers with pre- and post-protocol assessments. All 32 implanted patients were included in adverse event reporting. Participant flow, including reasons for exclusion, is detailed in Figure 2.

**Figure 2:**
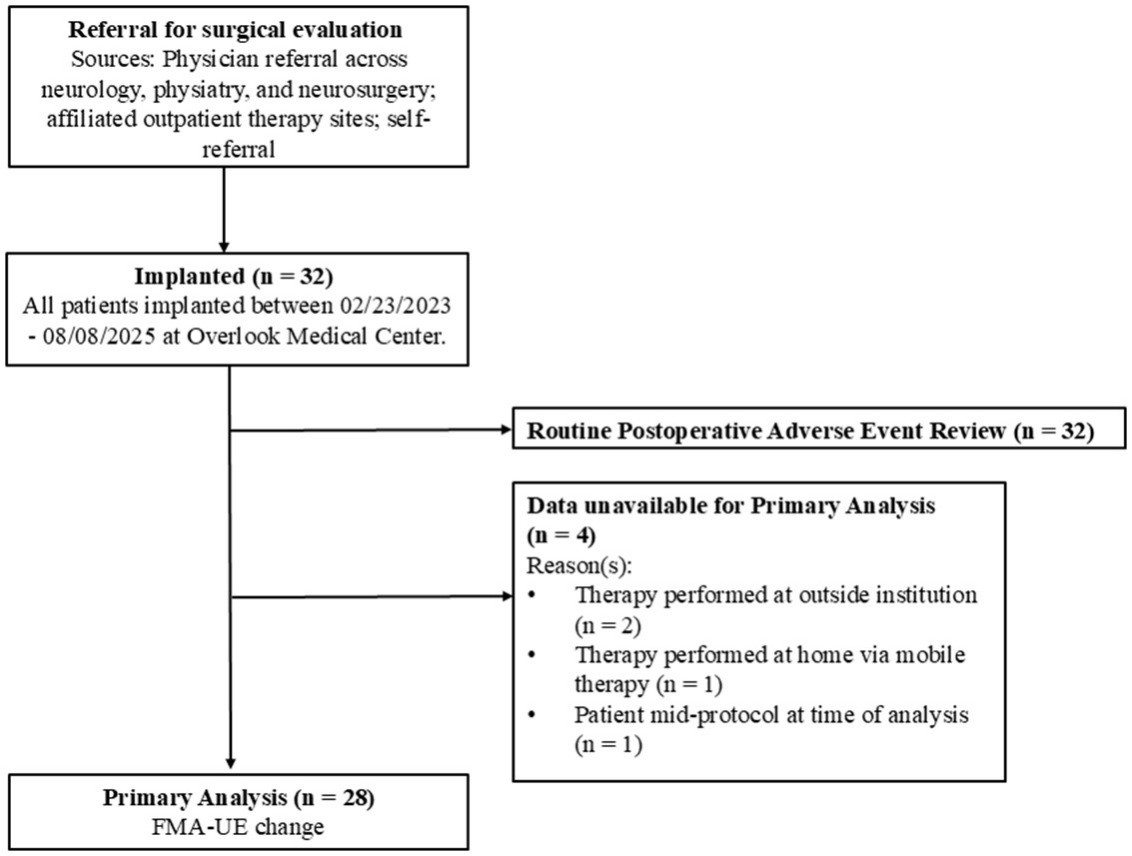
STROBE flow diagram. Flow of patients through the retrospective cohort. Thirty-two patients underwent device implantation and were included in adverse-event reporting. Twenty-eight patients completed the intended Paired VNS protocol within the health system and had paired baseline and end-of-protocol Fugl-Meyer Assessment–Upper Extremity (FMA-UE) scores available for the primary analysis.

### 3.2 Demographics

Ages ranged from 34 to 81 years (mean 62.1; SD 12.4). In the full cohort (n = 32), the median time since stroke was 3.0 years (IQR 1.7–4.2; range 1.0–11.0; M = 3.4, SD = 2.2); among the 28 patients included in the primary analysis, the median was 3.0 years (IQR 1.7–4.0; range 1.0–11.0; M = 3.1, SD = 2.0). Baseline FMA-UE scores were available for 31 patients (mean 34.2; SD 11.1; median = 34; IQR 25.5–46.5; range 17–54); the score was unavailable for the patient who experienced seizures and was treated outside the system. Impairment severity across the 31 patients with available baseline FMA-UE scores was concentrated in the two middle categories of a published four-level FMA-UE severity classification (19) with 17 (54.8%) in the severe-moderate range and 13 (41.9%) in the moderate-mild range. One patient scored in the mild range and there were no patients in the severe category. Available characteristics for the full cohort and for the included and excluded subgroups are summarized in Table 1.

**Table 1:** Baseline characteristics.

Variable	Full cohort (*n* = 32)	Included in primary analysis (*n* = 28)	Excluded from primary analysis (*n* = 4)
Sex, *n* (%)			
Male	17 (53.1)	15 (53.6)	2 (50.0)
Female	15 (46.9)	13 (46.4)	2 (50.0)
Age, years			
Mean (SD)	62.1 (12.4)	63.0 (12.8)	56.3 (7.4)
Median (IQR)	65.5 (55.3–69.3)	67.5 (56.0–70.0)	56.0 (51.8–60.5)
Range	34–81	34–81	48–65
Time since stroke, years			
Mean (SD)	3.4 (2.2)	3.1 (2.0)	5.2 (3.1)
Median (IQR)	3.0 (1.7–4.2)	3.0 (1.7–4.0)	5.5 (4.1–6.6)
Range	1.0–11.0	1.0–11.0	1.0–8.6
FMA-UE^a^			
n	31	28	3
Mean (SD)	34.2 (11.1)	33.4 (11.0)	41.3 (11.0)
Median (IQR)	34.0 (25.5–46.5)	32.0 (24.5–46.3)	36.0
Range	17–54	17–53	34–54
Impairment level, *n* (%)			
Severe (0–15)	0 (0)	0 (0)	0
Severe-moderate (16–34)	17 (54.8)	16 (57.1)	1
Moderate-mild (35–53)	13 (41.9)	12 (42.9)	1
Mild (54–66)	1 (3.2)	0 (0)	1

Available baseline characteristics stratified by inclusion vs. exclusion from the primary analysis. No formal statistical comparison between subgroups was performed given the small number of excluded patients. FMA-UE, Fugl-Meyer Assessment–Upper Extremity (range 0–66; higher scores indicate less motor impairment); IQR, interquartile range; SD, standard deviation.

a Baseline FMA-UE was unavailable for one patient, who experienced seizures and was treated outside the health system; denominators for this variable are therefore 31 and 3 (excluded), rather than 32 and 4. Baseline scores for the 3 excluded patients with available data were 34, 36, and 54. IQR and percentages are not reported for this subgroup given n = 3.

### 3.3 Upper extremity function

Among 28 local completers, the mean FMA-UE scores increased from 33.43 (SD, 11.01) at baseline to 42.86 (SD, 11.37), representing a mean within-participant change of 9.43 points (SD = 5.76; 95% CI = 7.20–11.66), t(27) = 8.67, *p* < 0.001. The median change was 8.0 points (IQR 5.0–13.25; range 1–25), and the difference scores did not depart significantly from normality (Shapiro–Wilk W = 0.937, *p* = 0.092). Twenty of 28 patients (71.4%; 95% CI 52.9%−84.7%) met or exceeded the 6-point MCID threshold for clinically meaningful improvement, and the remaining 8 patients improved by 1 to 5 points. The standardized magnitude of within-participant change was *d*_*z*_ = 1.64, presented as a descriptive summary of the observed change relative to its variability rather than as an estimate of treatment effect size. A 10,000-replicate percentile bootstrap of the mean difference yielded a 95% CI of [7.39, 11.61], consistent with the parametric estimate.

In descriptive analyses by baseline impairment severity, mean FMA-UE change was 10.6 points (SD, 6.4; 95% CI 7.19–14.06; median 10.5, IQR 5.75–14.25; range 1–25) among the 16 patients in the severe–moderate group and 7.8 points (SD, 4.5; 95% CI 4.99–10.67; median 7.0, IQR 5.00–8.25; range 2–17) among the 12 patients in the moderate–mild group. Responder rates were 75.0% (12/16; 95% CI 50.5%−89.8%) and 66.7% (8/12; 95% CI 39.1%−86.2%), respectively. These subgroup results are descriptive; no formal between-group comparisons were performed. Individual FMA-UE scores at baseline and end-of-protocol are presented in Figure 3, and individual changes are shown in Figure 4.

**Figure 3:**
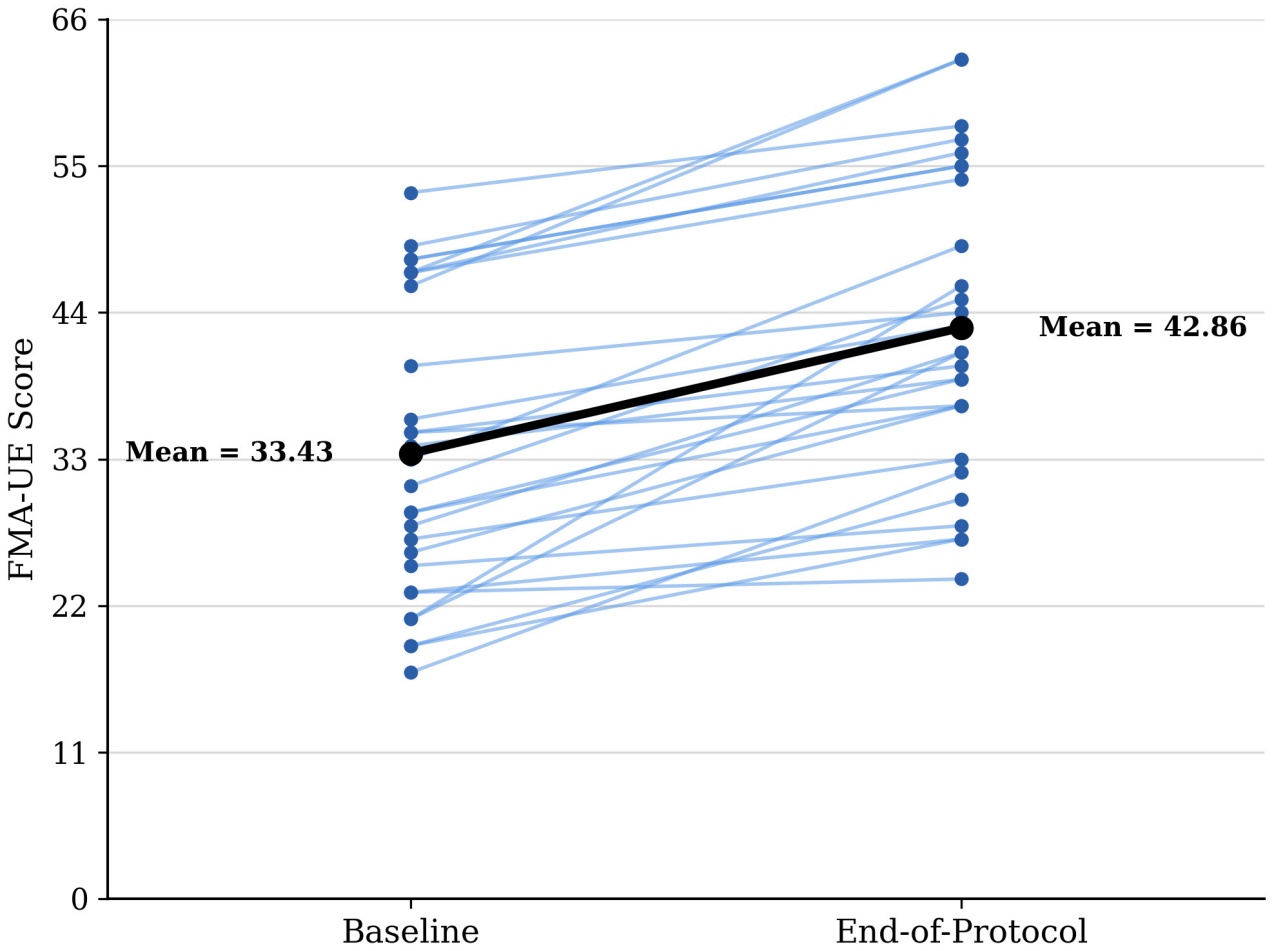
Individual and mean FMA-UE Scores. Paired baseline and end-of-protocol Fugl-Meyer Assessment–Upper Extremity (FMA-UE) scores for the 28 patients included in the primary analysis. Individual patient observations are connected across time, and the bold line represents the group mean.

**Figure 4:**
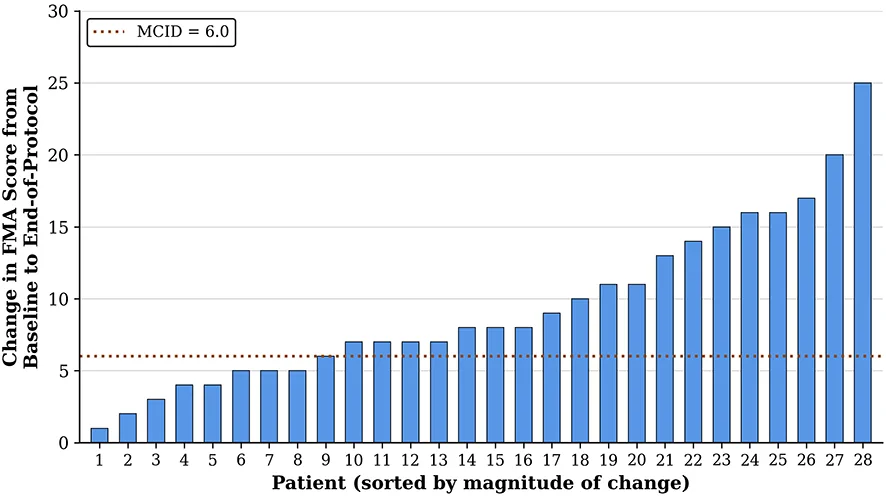
Change in FMA-UE scores. Individual changes in Fugl-Meyer Assessment–Upper Extremity (FMA-UE) score from baseline to the end of the Paired VNS protocol among the 28 patients included in the primary analysis. Change scores are presented in ascending order. The horizontal reference line indicates the minimum clinically important difference (MCID) threshold of ≥6 points.

Three patients chose to continue rehabilitation services beyond the intended protocol and had long-term follow-up data ranging from 5 to 22 months. Their FMA-UE assessment scores are presented here as exploratory case-level observations. Notably, while these individuals did not reach the MCID during the primary analysis period, all showed continued improvement and progress toward individual goals across subsequent assessments. Table 2 shows their additional FMA-UE assessments across available time points.

**Table 2:** Long-term FMA-UE scores.

Intended protocol (18 sessions)			Long-term follow-up				
Baseline FMA-UE^a^	End-of-protocol FMA-UE	Within-protocol change	Time since baseline, months	FMA-UE at follow-up	Post-protocol change	Total change from baseline	MCID met?^b^
35	37	+2	5	41	+4	+6	Yes
25	28	+3	14	49	+21	+24	Yes
23	27	+4	22	33	+6	+10	Yes

Exploratory long-term FMA-UE scores for the three participants who continued rehabilitation beyond the intended 18-session protocol. None of the three met the 6-point MCID threshold by the end of the protocol; all three met or exceeded it by their long-term assessment. These observations are case-level and exploratory and were not formally analyzed. FMA-UE, Fugl-Meyer Assessment–Upper Extremity (range 0–66; higher scores indicate less motor impairment); MCID, minimal clinically important difference.

a Baseline refers to initiation of Paired VNS therapy.

b Whether total change from baseline to long-term follow-up met or exceeded the 6-point MCID threshold.

### 3.4 Patient-reported and occupational therapist-observed changes

Clinical documentation provided additional context for the changes experienced among participants. Chart-derived clinical notes were available for 26 of the 28 analyzed patients and included OT observations as well as patient or caregiver-reported changes documented during Paired VNS sessions. Notes were not available for two participants at the time of the analysis. Six cases were purposively selected to represent a range of ages, baseline impairment levels, within-protocol FMA-UE changes, and clinically meaningful response status (available cases are provided as Supplementary material). These cases are presented in Table 3 to illustrate individual experiences alongside quantitative measures; they were not formally analyzed.

**Table 3:** Patient-reported and therapist-observed changes.

Time since stroke	End-of-protocol FMA-UE change	MCID met?^a^	Observed changes (summarized)	Source
2.7 years	+4	No	Able to write name and knit with the affected hand.	Therapist-observed and patient-reported
1.0 years	+14	Yes	Increased independence at home with improved fine motor control, writing, cutting food, ability to pick up small objects, and improved overhead reach.	Therapist-observed and patient-reported
4.0 years	+5	No	Improved extension at the wrist and all digits, with grip strength increased from 10 to 17 lb. Able to assist in daily activities including folding laundry, carrying objects, and meal preparation, with improved handwriting.	Therapist-observed and patient-reported
4.0 years	+3	No	Reports driving better and going fishing for the first time since stroke, casting approximately 75 feet.	Patient-reported
2.8 years	+8	Yes	Able to cut food with a knife and use a fork with a built-up handle. Reports improved activity tolerance and movement quality.	Patient-reported
4.8 years	+10	Yes	Reports improved ability to engage the affected hand during tasks such as applying deodorant, closing the microwave, sweeping, making the bed, preparing snacks, steering a shopping cart, opening doors, carrying trays, zipping bags, and completing laundry. Demonstrates improved extension at the wrist and third and fifth digits.	Therapist-observed and patient-reported

Patient-reported and therapist-observed changes following Paired VNS therapy, extracted from clinical notes. Cases were purposively selected to illustrate a range of within-protocol FMA-UE changes and response status; they do not represent formal measures of activity or participation. FMA-UE, Fugl-Meyer Assessment–Upper Extremity; MCID, minimal clinically important difference.

a Whether the within-protocol change met or exceeded the 6-point threshold.

Where appropriate, OTs collected additional material to visually track progress toward patient goals. For example, one individual who was 2.7 years post-stroke improved by 4 points on the FMA-UE. Although this change did not reach the 6-point MCID threshold, the patient achieved a personal therapy goal of improving penmanship sufficiently to write birthday cards to family members. Handwriting samples are presented in Figure 5 with the patient's permission.

**Figure 5:**
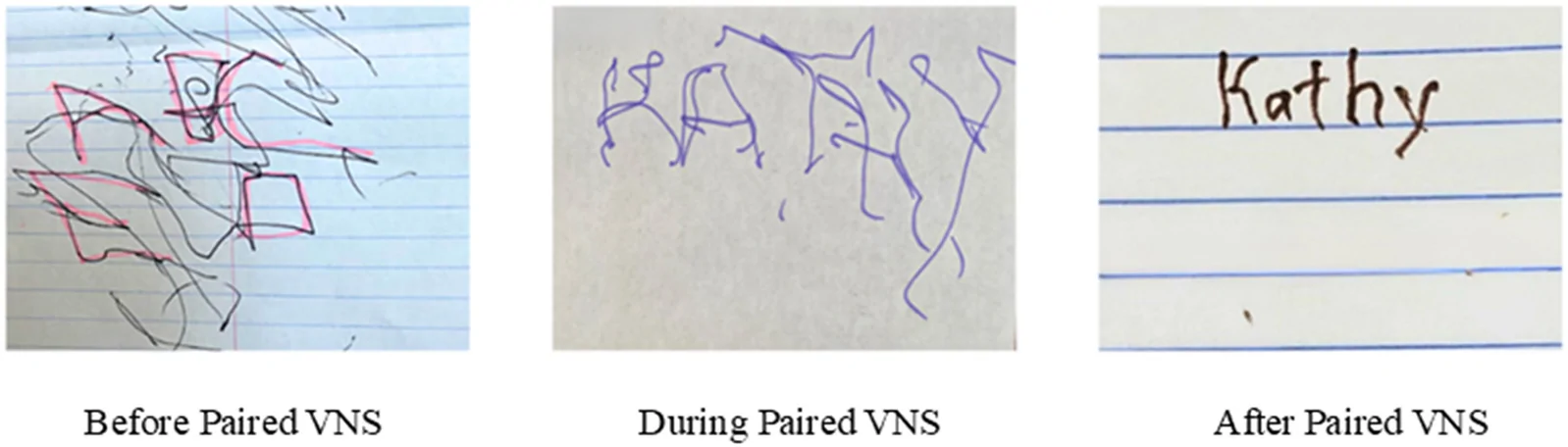
Example of meaningful individual goal. Illustrative handwriting samples obtained before, during, and after the Paired VNS protocol from a participant who was 2.7 years post-stroke. The samples are presented as illustrative clinical context. Published with the participant's permission.

### 3.5 Adverse events

All 32 patients underwent successful outpatient implantation. One patient developed a minor postoperative hematoma that resolved without intervention. A second patient elected to have the device removed after 410 days due to tingling on the chest. Because adverse events beyond routine early postoperative follow-up were captured through available clinical documentation rather than systematic surveillance, long-term incidence and relatedness cannot be estimated from this study.

## 4 Discussion

In this retrospective cohort of adults with chronic ischemic stroke, FMA-UE scores improved in every patient who completed the Paired VNS protocol within our health system and had paired assessments. Although our program did not apply a fixed FMA-UE threshold for candidacy, the baseline of all local completers (17–53) fell within the severe-moderate and moderate-mild impairment categories, with none classified in the severe or mild categories (19). The mean within-participant change was 9.43 points (95% CI 7.20–11.66), and 20 of 28 patients (71.4%) met or exceeded the defined 6-point clinically meaningful response threshold. Because this study evaluated routine clinical care, these results reflect the changes observed over a course of Paired VNS therapy in an uncontrolled environment rather than an isolated treatment effect. Although fundamental differences in study design limit direct comparison, the direction of change was consistent with findings from the VNS-REHAB trial (9) and published real-world cohorts reporting mean FMA-UE gains of 8.9 and 10.8 points (17, 21).

The analyzed cohort spanned from 1 to 11 years after the index stroke, and FMA-UE improvement was observed across the full range. Prior studies have also reported FMA-UE improvements among participants treated in the chronic phase, though differences in study design and patient selection limit direct comparison (10, 12, 13). Long-term clinical trial follow-up has found that some patients experience additional gains over time, with benefits sustained to 3 years (9, 12, 13, 15). Within this cohort, one patient who was implanted 11 years post-stroke showed the largest individual FMA-UE improvement. While a single observation should be considered anecdotal, it documents improvement in a patient despite long-standing impairment. In three patients with extended follow-up (5–22 months), additional motor improvement and progression toward individual goals was documented beyond the typical therapy protocol. These observations are exploratory and represent a small subset of the cohort.

Beyond the FMA-UE findings, clinical documentation provided illustrative context regarding changes in daily activities discussed or observed during therapy, including feeding and dressing, household management, and a return to hobbies. These chart-derived notes do not reflect standardized measures of activity and participation; nevertheless, they provide insight into the types of changes that patients, caregivers, or therapists identified as relevant during treatment with Paired VNS.

Implantation was successful in all 32 patients. One patient developed a minor postoperative hematoma that resolved without intervention, and another elected explantation 410 days after implantation because of chest tingling. No other adverse events were identified in the available clinical record. Because ascertainment relied on routine documentation during early postoperative follow-up and was not systematic thereafter, the long-term incidence and relatedness of events cannot be estimated.

Although this study did not formally evaluate implementation, our institutional experience identified several practical considerations that may affect program delivery and warrant future evaluation in other settings. Many individuals with chronic stroke are no longer engaged in follow-up care, and awareness of Paired VNS varies among patients and clinicians. Delivery requires coordination between surgical and rehabilitation teams, therapist training, and sufficient resources for high-repetition therapy and longer treatment sessions. Administrative requirements, including prior authorization, may also delay or limit treatment initiation. These considerations are particularly relevant in light of recent guidance from the American Heart Association/American Stroke Association calling for expanded access to post-acute stroke rehabilitation and the integration of patient-centered goals across the continuum of care (22). Future implementation research should evaluate barriers and identify strategies to support consistent patient identification, referral pathways, coverage development, and consistent program delivery.

### 4.1 Limitations

Several limitations should be acknowledged. First, the retrospective observational design, absence of a control group, and relatively small sample size limit causal inference and the precision of estimates. Selection occurred during referral and treatment, and the primary analysis was restricted to patients who completed the local protocol and had paired assessments. Second, as this study evaluated routine clinical care, OTs who delivered the intervention also performed outcome assessments. The lack of blinded assessment and formal evaluation of inter-rater reliability introduces the potential for expectation bias, and the observed changes may therefore differ from those estimated in controlled clinical trials. Third, clinical documentation was incomplete for some variables, including referral pathways, therapy dose, home self-activation, and selected patient characteristics, limiting assessment of potential selection bias, protocol adherence, and comparisons between included and excluded participants. Finally, this was a single-health-system experience in which all implantations were performed by one experienced neurosurgeon and rehabilitation was delivered by therapists with specialized Paired VNS training, which may limit generalizability to other institutions and practice settings.

## 5 Conclusion

This study describes the delivery of a multidisciplinary Paired VNS program across 5 outpatient clinics within the Atlantic Health System. Among the 28 patients who completed the intended therapy protocol locally, FMA-UE scores improved in every patient. The mean increase of 9.43 points was statistically significant (*p* < 0.001), and 20 patients (71.4%) met or exceeded the defined ≥6-point clinically meaningful response threshold. Selected chart-derived notes also provided illustrative context regarding changes in valued daily activities and hobbies that accompanied FMA-UE improvement. The observed FMA-UE findings were directionally consistent with those reported in prior randomized trials and observational studies of Paired VNS for UE recovery in chronic ischemic stroke. Future work should evaluate strategies to optimize program delivery, characterize access and referral barriers, and refine patient selection. Large-scale, longitudinal registries incorporating standardized measures of impairment, activity, participation, and therapy delivery will help describe outcomes across broader clinical populations and inform future evaluation of Paired VNS program delivery and implementation.

## Data Availability

The datasets presented in this article are not readily available because they contain information derived from clinical records that is subject to institutional and privacy protections. Access to a limited dataset may be considered upon reasonable request, subject to institutional approval, execution of an appropriate data-use agreement, and applicable privacy protections. Requests to access the datasets should be directed to Ronald P. Benitez, ronald.benitez@atlanticbrainandspine.org.
