# Supplementary material for: Upper extremity motor outcomes during paired vagus nerve stimulation in chronic ischemic stroke: a retrospective single-health-system cohort study
Source: Front Neurol. 2026 Sep 30;17:1872498. doi: 10.3389/fneur.2026.1872498 (PMC13661468; doi:10.3389/fneur.2026.1872498)
Supplement: Supplementary file 1 [file Table_1.doc]

# Supplemental Table. Individual Outcomes and Clinical Notes

*All implanted cases are listed for transparency. Cases 15, 22, 25, and 26 (shaded) were excluded from the primary FMA-UE analysis (n = 28) as noted; Case 15 was still undergoing therapy at the time of analysis. Cases 22, 25, and 26 completed the protocol at an outside health system and outcome data was unavailable. Cases 10 and 13 had no end-of-protocol notes available. Summaries of therapist notes were derived from clinical documentation and may include subjective reports from the patient or family member rather than direct therapist observation or objective measurement.*

| **Patient** | **Time Since Stroke (yrs)** | **Baseline FMA-UE** | **End-of-Protocol FMA-UE** | **Summary of Therapist Notes** |
| --- | --- | --- | --- | --- |
| 1 | 3.0 | 19 | 30 | Improved reaching, hand-to-mouth function, attempted drinking from a bottle, and improved resting & passive extension of the Right 3rd to 5ᵗʰ digit. Residual hand tone remained limiting though has improved. |
| 2 | 1.1 | 49 | 57 | Planned to continue therapy using the magnet. Reported ability to perform tasks that were previously not possible. |
| 3 | 4.2 | 28 | 41 | Reported improved hand opening and increased compliance with resting hand splint; finger extension remained limited by tone/spasticity. |
| 4 | 3.3 | 27 | 33 | Reported improvements in speech, strength, dressing, pinch function, self-feeding, and ability to feed the family dog. |
| 5 | 1.9 | 47 | 54 | Increased spontaneous use of the affected arm during daily activities (from approximately 10% to 80%). Improved upper-body dressing and beginning buttoning tasks. |
| 6 | 4.0 | 35 | 40 | Improved wrist/finger extension and grip strength (10 to 17 lb). Improved meal preparation, laundry, carrying objects, handwriting, and hand opening. |
| 7 | 1.1 | 29 | 37 | Improved shoulder ROM, pinch strength, grip strength, and upper-extremity mobility reported by spouse. |
| 8 | 1.0 | 31 | 45 | Improved writing, fine motor control, finger movement, dexterity, food preparation, overhead reaching, and independence at home. Patient demonstrates improved word finding and speech. |
| 9 | 2.8 | 46 | 63 | Reported smoother movement and improving typing ability; able to perform tasks previously not possible. |
| 10 | 1.8 | 34 | 39 | Not documented. |
| 11 | 1.4 | 26 | 37 | Marked distal motor improvement with improved household activities including clearing the table, washing dishes, and making the bed. |
| 12 | 1.4 | 47 | 56 | Increased spontaneous arm use. Easier dressing, eating, shoe tying, driving, and jar opening. Continued goals included typing and makeup application. |
| 13 | 3.6 | 47 | 63 | Not documented. |
| 14 | 2.7 | 40 | 44 | Achieved personal goals of writing name and knitting with the affected hand. Demonstrated isolated voluntary digit movement. |
| 15 | 5.1 | 36 | — | *Mid-protocol at time of data cutoff; excluded from the primary FMA-UE analysis.* |
| 16 | 1.0 | 33 | 49 | Reported improvements in walking, balance, food preparation, manipulation of small objects, and urinary continence. Continued outpatient therapy. |
| 17 | 3.0 | 36 | 43 | Improved wrist extension, supination, fine motor control, balance, self-feeding, and progress toward return to golf. |
| 18 | 1.3 | 23 | 24 | Improved gait, endurance, confidence with ambulation, and sit-to-stand performance. |
| 19 | 4.2 | 48 | 55 | Therapy completion was delayed by 1 week because of influenza. |
| 20 | 4.8 | 23 | 27* | Increased wrist/digit activation and greater use of the affected arm during meal preparation, shopping, laundry, door opening, work tasks, and other daily activities. |
| 21 | 4.8 | 29 | 39 | Improved self-feeding, cutting food, and deodorant application. Continued goal of returning to photography. |
| 22 | 1.0 | — | — | *Completed the protocol at an outside health system; complete FMA-UE data were unavailable.* |
| 23 | 2.6 | 35 | 37* | Returned to playing piano for the first time since her stroke. Achieved independent lower-body dressing, including socks, shoes, and pants. Improved ability to reach the top of the head and behind the back. |
| 24 | 2.8 | 19 | 27 | Improved grasp/release, food cutting, activity tolerance, gait quality, and mild speech improvement. |
| 25 | 5.9 | 34 | — | *Completed the protocol at an outside health system; end-of-protocol FMA-UE data were unavailable.* |
| 26 | 8.6 | 54 | — | *Completed the protocol at an outside health system; end-of-protocol FMA-UE data were unavailable.* |
| 27 | 4.3 | 48 | 55 | Improved dexterity and handwriting despite persistent sensory impairment. Continued home practice. |
| 28 | 4.0 | 25 | 28* | Reported improved wrist/finger extension, driving, and return to fishing. Continued home therapy with magnet use. |
| 29 | 3.1 | 21 | 41 | Decreased tone and improved wrist/finger ROM, active finger extension, and endurance. |
| 30 | 3.3 | 17 | 32 | Continued 90-minute therapy sessions because of ongoing gains. Marked improvement in hand and wrist function with residual shoulder weakness. |
| 31 | 4.8 | 53 | 58 | Improved fine motor control, finger movement, coordination, and ability to play guitar. |
| 32 | 11.0 | 21 | 46 | Functional goals included gardening, mowing, dressing, and household activities. Demonstrated substantial motor recovery despite chronicity. |

** Participant continued Paired VNS beyond the intended protocol for the primary analysis period; additional follow-up FMA-UE data are presented in Table 2.*
